# Critical role of actin-associated proteins in smooth muscle contraction, cell proliferation, airway hyperresponsiveness and airway remodeling

**DOI:** 10.1186/s12931-015-0296-1

**Published:** 2015-10-30

**Authors:** Dale D. Tang

**Affiliations:** Center for Cardiovascular Sciences, Albany Medical College, 47 New Scotland Avenue, MC-8, Albany, NY 12208 USA

## Abstract

Asthma is characterized by airway hyperresponsiveness and airway remodeling, which are largely attributed to increased airway smooth muscle contractility and cell proliferation. It is known that both chemical and mechanical stimulation regulates smooth muscle contraction. Recent studies suggest that contractile activation and mechanical stretch induce actin cytoskeletal remodeling in smooth muscle. However, the mechanisms that control actin cytoskeletal reorganization are not completely elucidated. This review summarizes our current understanding regarding how actin-associated proteins may regulate remodeling of the actin cytoskeleton in airway smooth muscle. In particular, there is accumulating evidence to suggest that Abelson tyrosine kinase (Abl) plays a critical role in regulating airway smooth muscle contraction and cell proliferation in vitro*,* and airway hyperresponsiveness and remodeling in vivo. These studies indicate that Abl may be a novel target for the development of new therapy to treat asthma.

## Introduction

Airway smooth muscle cell contraction and proliferation contribute to the pathogenesis of airway hyperresponsiveness (AHR) and airway remodeling [1–8], cardinal features of asthma that affect nearly 25 million people in the United Sates and 250 million people worldwide (www.cdc.gov and www.who.int). However, the cellular and molecular mechanisms that regulate smooth muscle cell contraction and proliferation are not fully understood. Understanding these cellular processes is fundamental to our knowledge regarding smooth muscle biology and the pathogenesis of asthma.

Upon external stimulation, myosin light chain undergoes phosphorylation at Ser-19, which activates myosin ATPase and initiates sliding of contractile filaments and smooth muscle contraction [9, 10]. More importantly, there is a wealth of evidence to suggest that actin cytoskeletal remodeling is critical for smooth muscle contraction. A pool of actin monomers polymerizes onto existing actin filaments in smooth muscle in response to contractile stimulation. Inhibition of the G-actin to F-actin transition by pharmacological tools and molecular approach attenuates smooth muscle contraction without affecting myosin light chain phosphorylation [11–14]. Actin filament polymerization may promote smooth muscle force development by enhancing the transmission of force between the contractile unit and the extracellular matrix [11–16]. Smooth muscle contraction is similar to “the moving of a car”. Myosin may serve as an “**engine**” for smooth muscle contraction whereas the actin cytoskeleton may function as a “**transmission system**” in smooth muscle [17, 18].

During breathing, airway smooth muscle is constantly subjected to mechanical oscillation, which affects airway smooth muscle contractility. There is evidence that mechanical stretch induces fluidization and resolidification of smooth muscle cells, suggesting actin cytoskeletal remodeling (actin depolymerization and polymerization) in the cells upon transient stretch [19]. However, the mechanisms underlying the dynamic actin cytoskeleton are not well elucidated.

The mitogen-activated protein kinase (MAPK) pathway plays an essential role in regulating various cellular functions including cell proliferation [20–22]. In response to stimulation with growth factors within minutes, MEK1/2 (MAPK kinase) gets phosphorylated by Raf-1 kinase [22, 23], which in turn phosphorylates and activates extracellular signal-regulated kinase1/2 (ERK1/2). Activated ERK1/2 phosphorylates several protein kinases, transcription factors, and other proteins to promote cell proliferation eventually [20–23]. Recent studies demonstrate a critical role of actin-regulatory proteins in the growth factor-associated signaling and proliferation in smooth muscle cells [20, 21, 24].

This review will summarize our current understanding of physiological properties of the actin-associated proteins in smooth muscle reactivity and proliferation in vitro and their roles in the pathogenesis of AHR and airway remodeling in vivo. In particular, there is evidence to suggest that Abelson tyrosine kinase (Abl, c-Abl) plays a critical role in controlling airway smooth muscle contraction and cell proliferation in vitro*,* and AHR and airway remodeling in vivo. These studies indicate that Abl may be a novel target for the development of new therapy to treat asthma.

## Role of actin-associated proteins in smooth muscle contraction

### Critical role of actin polymerization in smooth muscle contraction

Actin filaments of smooth muscle cells attach to dense plaques containing integrins on the membrane, and connect with dense bodies in the cytoplasm. The majority of actin (thin) filaments localize around myosin (thick) filaments in a rosette array, forming the contractile machinery. This pool of actin filaments is referred to as “contractile actin”. In addition, “cytoskeletal actin” does not structurally interact with myosin filaments, which plays a role in maintaining the structural integrity of smooth muscle cells. The structure of the actin cytoskeleton in smooth muscle cells has been described in detail elsewhere [14, 25, 26].

#### Actin polymerization and depolymerization occur during the contraction-relaxation cycle of smooth muscle

There is accumulating evidence to suggest that actin filament polymerization transpires in smooth muscle in response to contractile activation, and actin depolymerization occurs during the relaxation of smooth muscle. First, we and others have used an actin fractionation assay and calculated that in unstimulated airway smooth muscle cells/tissues, 70–80 % of total actin exists in insoluble actin (F-actin) whereas 20–30 % of total actin is found in soluble actin (G-actin). In response to contractile activation, 85–90 % of total actin is F-actin in airway smooth muscle cells/tissues [11, 16–18, 27–30]. By using the fractionation assay, a number of research teams have found that contractile stimulation increases the amount of F-actin and/or decreases the G-actin level in arterial smooth muscle tissues [12, 13, 29–31]. Furthermore, the F-actin/G-actin ratios are reduced in smooth muscle during relaxation [31–33]. Second, several groups including our laboratory have also used fluorescent microscopy to evaluate F-actin and G-actin by reacting smooth muscle tissues/cells with phalloidin (for F-actin) and DNase I (for G-actin) conjugated with fluorescent labels [33–36]. Treatment of human airway smooth muscle cells with carbachol and endothelin-1 leads to the increase in the ratio of F-actin to G-actin as estimated by fluorescence microscopy, whereas treatment with smooth muscle relaxants (isoproteronol and forskolin) decreases the F-actin/G-actin ratios [33, 36, 37]. In other studies, stimulation with vasoconstrictors enhances the F-actin/G-actin ratios in vascular smooth muscle as evaluated by fluorescent microscopy [12, 34, 35]. Third, a decrease of the G-actin pool was observed in contractile airway smooth muscle tissues by using the DNase inhibition assay [11, 14]. In addition, Barany et al. also documented a reduced G-actin content in contractile arterial smooth muscle by measuring the exchange rates of actin-bound nucleotide [11, 38]. Taken together, these studies demonstrate that actin polymerization and depolymerization occur in various smooth muscle cell/tissue types during the contraction-relaxation cycle.

#### Inhibition of actin polymerization attenuates smooth muscle contraction

The inhibition of actin polymerization by pharmacological agents such as cytochalasin or latrunculin attenuates the contraction in a variety of smooth muscle tissues including airway smooth muscle [32, 35, 39–41]. Cytochalasin caps existing actin filaments at the barbed end, preventing F-actin elongation whereas latrunculin binds to G-actin and blocks the assembly of G-actin onto actin filaments [11, 14, 42, 43]. The short-term treatment of smooth muscle with these inhibitors does not impair the organization and ultrastructure of contractile filaments [11, 14]. Furthermore, inhibition of actin polymerization by molecular approaches that interfere with actin-regulatory proteins also attenuates smooth muscle contraction [16–18, 30, 35, 44–48]. More importantly, the suppression of actin polymerization inhibits force development in smooth muscle with little or no effects on myosin light chain phosphorylation, a cellular event that is essential for smooth muscle contraction [16–18, 30, 35, 44–48]. Therefore, these results suggest that actin filament polymerization and myosin activation are two parallel cellular processes that are fundamental for the regulation of smooth muscle contraction.

How does actin polymerization affect the contraction in smooth muscle? First, the actin filaments of smooth muscle cells connect to the cytoplasmic domain of β integrins via linker proteins such as vinculin, talin and α-actinin whereas the extracellular portion of β integrins engages with the extracellular matrix [11, 14, 15, 25, 26, 49, 50]. Thus, the actin-integrin-matrix connection provides the structural basis for the force transmission between the contractile unit and the extracellular matrix [11, 14, 15, 25, 26, 49–53]. Nascent actin polymerization may occur at the cell cortex of smooth muscle [11, 14, 15, 25, 27, 28, 49, 54]. Cortical actin assembly may strengthen the linkage of actin filaments to integrins and enhance the transmission of contractile force [11, 14, 15, 25, 30, 35, 44, 53, 55].

Second, actin filament assembly may participate in the “latch” formation of contractile filaments, supporting force maintenance under the condition of lower crossbridge phosphorylation [13, 14, 56, 57]. Third, our recent studies suggest that actin polymerization promotes the recruitment of β-catenin to N-cadherin (critical components of intercellular junctions), which may facilitate the cell-to-cell force transmission and contraction [58].

### Regulation of actin dynamics in smooth muscle

In the last decade, a wealth of information has been accumulated to elucidate how actin filament polymerization/depolymeration is regulated in smooth muscle. Thus far, protein kinases, such as Abl, p21-activated kinase (PAK), focal adhesion kinase (FAK), integrin-linked kinase (ILK) and other kinases, have been reported to regulate actin polymerization in smooth muscle. Transmembrane integrins may be able to activate signaling pathways coordinating actin dynamics in smooth muscle. Rho, Cdc42, and Rac are the major members of Rho family of the small GTPases that mediates actin polymerization in smooth muscle. The actin-regulatory proteins are effectors in the signaling cascades to mediate actin dynamics. Some of the proteins are neuronal Wiskott-Aldrich syndrome Protein (N-WASP), the Arp2/3 complex, cortactin, glia maturation factor-γ (GMF-γ), Abl interactor 1 (Abi1), profilin, cofilin, and heat shock proteins. Generally speaking, receptor activation and/or integrin ligation activates protein kinases and/or small GTPases, which subsequently regulate the functional status of the actin regulatory proteins and eventually actin filament assembly or structural reorganization. However, these protein interactions and signaling pathways are complex and may be cell-type and species dependent. The expression and role of actin-associated proteins in different cell types and species are summarized in Table 1.

**Table 1:** The expression and role of actin-associated proteins in different cell-types and species

Name	Cell types	Species	Role	References
Abi1	ASMCs, VSMCs	Human	Adapter protein	[17]
Abl	ASMCs, VSMCs	Human, rat, mouse	Tyrosine kinase	[5, 17, 47, 48]
Arp2/3	ASMCs, VSMCs	Human, rat, canine	Promote actin branching	[17, 48, 54]
CAS	ASMCs, VSMCs	Human, rat	Adapter protein	[17, 48]
Cdc42	ASMCs	Canine	Small GTPase	[11, 27, 28]
Cofilin	ASMCs	Canine	Actin regulator	[45]
Cortactin	ASMCs	Human	Adapter protein	[18, 125]
CrkII	ASMCs	Canine	Adapter protein	[27]
FAK	ASMCs	Canine	Tyrosine kinase	[87, 88]
GMF-γ	ASMCs	Human	Promote actin debranching	[16]
HDAC8	ASMCs	Human, mouse	Histone deacetylase	[111]
HSP27	ASMCs	Canine	Heat shock protein	[53]
ILK	ASMCs	Canine	Adapter protein, kinase	[11, 55]
Integrin-β_1_	ASMCs	Human, canine	Link ECM to actin cytoskeleton	[52, 55]
N-Cadherin	ASMCs	Human	Adherens junction protein	[58]
N-WASP	ASMCs, VSMCs	Human, rat, canine	Promote actin nucleation	[17, 48, 55]
p38MAPK	ASMCs	Canine	Serine/threonine kinase	[93]
PAK1	ASMCs	Canine, mouse, human	Serine/threonine kinase	[80, 93, 131]
Parvin	ASMCs	Canine	Actin binding protein	[55]
Paxillin	ASMCs	Canine	Adapter protein	[55, 82, 83]
PINCH	ASMCs	Canine	ILK binding partner	[11, 55]
Profilin	ASMCs, VSMCs	Human, canine	Promote actin transport	[18, 32]
RhoA	ASMCs	Canine, human	Small GTPase	[30, 104]
Talin	ASMCs	Canine	Structural protein	[29, 51]
VASP	ASMCs	Canine	Phosphoprotein	[44]
Vinculin	ASMCs	Canine	Structural protein	[29, 51]
α-Actinin	ASMCs	Canine	Cross linking protein	[52]
β-Catenin	ASMCs	Human	Adherens junction protein	[58]

*ASMCs* airway smooth muscle cells, *VASMCs* vascular smooth muscle cells

#### Role of Abl tyrosine kinase in smooth muscle

Abl is a non-receptor protein tyrosine kinase that is ubiquitously expressed and has been implicated to function in a variety of cellular processes including the regulation of the actin cytoskeleton that mediates cell migration and adhesion [59–61]. There is evidence that Abl tyrosine kinase is necessary for airway smooth muscle contraction. Smooth muscle cell–specific knockout of Abl attenuates contractile responses of mouse tracheal rings to acetylcholine (ACh) [5]. Moreover, acute treatment with the Abl pharmacological inhibitors imatinib (Gleevec, STI-571) [5, 47, 62] and GNF-5 [63] significantly inhibits force development in mouse tracheal rings induced by ACh. Furthermore, treatment with imatinib or GNF-5 induces the relaxation of tracheal rings precontracted by ACh [5]. In addition, treatment with imatinib attenuates airway smooth muscle reactivity *in vivo* [64].

Abl has also been shown to regulate vascular smooth muscle contraction. Knockdown of Abl inhibits force development in arterial smooth muscle tissues during contractile activation [48]. Moreover, treatment with a cell permeable peptide or imatinib attenuates vascular smooth muscle contraction [47]. Interestingly, administration of the Abl inhibitor imatinib lessens the clinical symptoms of patients with pulmonary arterial hypertension [65, 66], suggesting a role for Abl in pulmonary arterial contraction.

How does Abl regulate force development in smooth muscle? To answer the question, the effects of Abl knockdown or inhibition on actin dynamics and myosin phosphorylation was evaluated. Knockdown of Abl attenuates actin polymerization in smooth muscle cells/tissues during contractile stimulation [14, 17, 48]. Inhibition of Abl activation by a cell permeable peptide also diminishes actin dynamics in smooth muscle stimulated by contractile agonists [47]. However, silencing or inhibition of Abl does not affect myosin light chain phosphorylation in smooth muscle [47, 48]. These observations suggest that Abl regulate smooth muscle contraction by controlling actin polymerization, but not myosin activation during contractile stimulation [11, 12, 16–18, 35, 45].

Contractile stimulation induces phosphorylation of Abl tyrosine kinase at Tyr-412 in smooth muscle [47, 48]. Tyr-412 is located at the activation loop of Abl kinase domain. When unstimulated, the activation loop of the Abl kinase domain folds into the active site, thereby preventing binding of both the substrate and ATP. Phosphorylation at Tyr-412 induces conformation changes; the activation loop no longer blocks the active site, which leads the increase in kinase activity [14, 47, 48, 60]. Abl tyrosine phosphorylation is regulated by Src in smooth muscle cells/tissues in response to contractile activation [15, 48, 67]. Similarly, Abl phosphorylation is also mediated by Src in fibroblasts [68] and cancer cells [69].

**Fig. 1:**
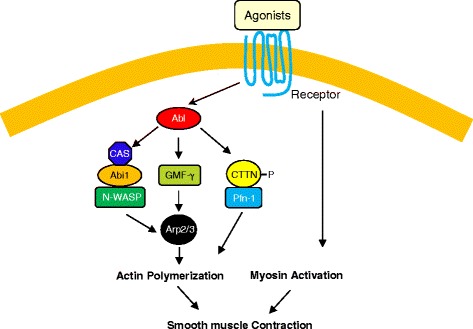
Mechanisms of Abl-regulated actin polymerization. Stimulation with agonists such as acetylcholine may activate Abl tyrosine kinase, which regulates the formation of the multiprotein complex including Crk-associated substrate (CAS), the adapter protein Abi1 (Abl interactor 1) and N-WASP (neuronal Wiskott-Aldrich Syndrome Protein), which in turn activates N-WASP, actin polymerization and smooth muscle contraction. Activated Abl also catalyzes phosphorylation of GMF-γ (glia maturation factor-γ) at Tyr-104, which induces the dissociation of GMF-γ from Arp2/3, and promotes actin polymerization. Furthermore, contractile agonists promote the association of cortactin (CTTN) with profilin-1 (Pfn-1), which induces actin polymerization and smooth muscle contraction. The interaction of cortactin with Pfn-1 is regulated by cortactin phosphorylation and Abl tyrosine kinase

Abl regulates actin dynamics in smooth muscle by controlling several downstream effectors. Abi1 is an adapter protein that has been implicated in the regulation of actin dynamics *in vitro* [70], cell adhesion and migration [71, 72]. In human airway smooth muscle cells/tissues, contractile stimulation induces an increase in the association of Abi1 with N-WASP (an actin nucleation activator) [17]. N-WASP is known to regulate the Arp2/3-mediated actin polymerization and branching [55, 73, 74]. Furthermore, contractile stimulation activates N-WASP in live smooth muscle cells as evidenced by changes in fluorescence resonance energy transfer efficiency of an N-WASP sensor [17]. Abi1 is necessary for N-WASP activation, actin polymerization and the contraction in smooth muscle. However, Abi1 does not affect myosin light chain phosphorylation [17]. More importantly, contractile activation induces the formation of a multiprotein complex including Abl, Crk-associated substrate (CAS) and Abi1. CAS is an adapter protein that participates in the regulation of smooth muscle tension development [15, 32, 39, 67]. Knockdown of Abl and CAS attenuates the activation of Abi1 during contractile activation. Collectively, the results indicate that Abl tyrosine kinase is able to regulate the formation of the CAS/Abi1/N-WASP complex, which subsequently activates N-WASP and actin polymerization [15, 17, 32, 39] (Fig. 1).

Cortactin is an actin-regulatory protein that is able to regulate actin filament assembly in *in vitro* studies as well as adhesion, migration, endocytosis of nonmuscle cells [75, 76]. In human airway smooth muscle cells/tissues, cortactin controls actin polymerization and force development without affecting myosin activation [18]. Furthermore, contractile stimulation induces cortactin phosphorylation at Tyr-421 and the association of cortactin with profilin-1 (Pfn-1) that is able to transport actin monomers onto actin filaments [18, 32, 39]. The cortactin/Pfn-1 complex is also rapidly recruited to the membrane upon contractile activation. The disruption of the protein-protein interaction by a cell permeable peptide attenuates actin polymerization and smooth muscle contraction. More importantly, Abl has a critical role in regulating the agonist-induced cortactin phosphorylation and the interaction of cortactin with Pfn-1 [18]. These results suggest that cortactin phosphorylation and cortactin/Pfn-1 coupling play a pivotal role in regulating actin dynamics and smooth muscle contraction. Abl regulates the association of cortactin with Pfn-1 and actin polymerization by catalyzing cortactin phosphorylation (Fig. 1).

GMF-γ is a member of actin-depolymerizing factor (ADF)/cofilin family that is widely expressed in eukaryotes and plays a central role in reorganizing the actin cytoskeleton by inducing actin network debranching [77, 78]. GMF-γ is able to inhibit actin nucleation *in vitro* [78]. Moreover, *in vitro* biochemical studies show that GMF-γ may induce debranching of the actin filament networks [77, 78]. Knockdown of GMF-γ enhances actin polymerization and the contraction in human airway smooth muscle cells/tissues with little or no effect on myosin phosphorylation [16]. Contractile activation induces GMF-γ phosphorylation at Tyr-104 and dissociation of GMF-γ from Arp2 of the Arp2/3 complex, which is regulated by Abl tyrosine kinase. Furthermore, expression of mutant Y104F GMF-γ attenuates actin polymerization and contraction in smooth muscle [16]. Thus, Abl tyrosine kinase is able to phosphorylate GMF-γ at Tyr-104, which inhibits the ability of GMF-γ to induce actin network disassembly (Fig. 1).

#### Role of the integrin-associated complex in actin polymerization

**Fig. 2:**
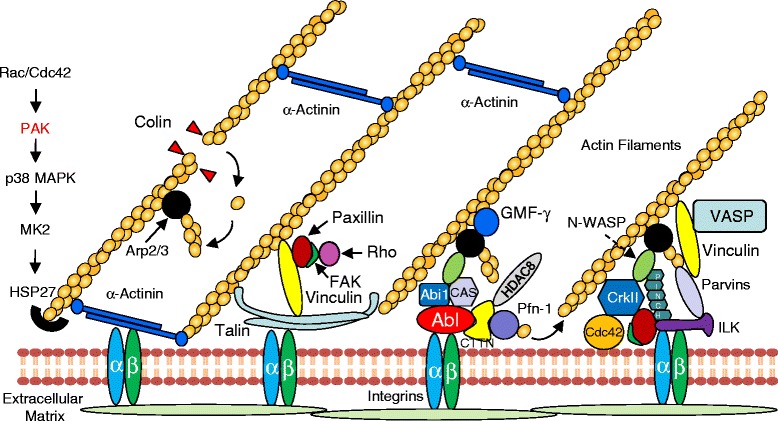
Molecular interactions of scaffolding/signaling proteins at or near intergrin-associated junctions. Cytoplasmic tails of integrins connect with actin filaments via actin linker proteins such as talin, vinculin, paxillin and α-actinin. The extracellular domains of integrins interact with the extracellular matrix, forming the integrin-associated junctions. Signaling and scaffolding proteins are assembled at or near the integrin-associated junctions upon contractile activation, which facilitates actin polymerization and the mechanotransduction between the contractile unit and the extracellular matrix. Abi1, Abl interactor 1; Abl, Abelson tyrosine kinase; CAS, Crk-associated substrates; CTTN, cortactin; FAK, focal adhesion kinase; GMF-γ, glia maturation factor-γ; HDAC8, histone deacetylase 8; HSP, heat shock protein; ILK, integrin-linked kinase; MAPK, mitogen-activated protein kinase; MK2, MAP kinase-activated protein (MAPKAP) kinase 2; N-WASP, neuronal Wiskott-Aldrich Syndrome Protein; PAK, p21-activated kinase; Pfn-1, profilin-1; VASP, vasodilator stimulated phosphoprotein

Transmembrane integrins are localized in membrane-associated dense plaques (similar to focal adhesion sites of cultured cells) and connect the extracellular matrix with the actin cytoskeleton in smooth muscle [11, 14, 15]. In addition, a number of structural and signaling proteins are found in the integrin-associated structure (Fig. 2). During cell migration, the engagement of integrins with the extracellular matrix initiates the recruitment of actin-associated proteins to the membrane, which eventually promotes actin polymerization [11, 61, 79]. There is evidence to suggest that contractile stimulation induces the assembly of the multiprotein complex at the membrane, which is critical for the regulation of actin polymerization in smooth muscle [30, 35, 44, 45, 47, 55, 80, 81].

In canine tracheal smooth muscle, a stable protein complex containing ILK (a β-integrin binding scaffolding protein and protein kinase), PINCH (an ILK binding partners) and α-parvin (an actin-binding protein) is recruited to integrin adhesion sites in response to contractile stimulation, where it interacts with β-integrins and forms a platform for the recruitment of other structural and signaling proteins that are required for the processes of actin cytoskeletal remodeling and mechanotransduction [11, 55].

Paxillin, a scaffolding and signaling protein, is required for tracheal smooth muscle contraction [82, 83] and is also recruited to membrane adhesion sites in response to contractile stimulation, where it associates with the ILK-associated complex [55]. Paxillin has been shown to undergo tyrosine phosphorylation in response to contractile stimulation in many smooth muscle cell and tissue types [13, 84–86]. The tyrosine phosphorylation of paxillin at Tyr-31 and −118 promotes its interaction with the SH2/SH3 adaptor protein CrkII, which binds to N-WASP and contributes to N-WASP and Arp2/3 activation [27]. Paxillin phosphorylation is mediated by FAK in smooth muscle [87, 88].

Integrin activation may also stimulate FAK and paxillin in smooth muscle; the tyrosine phosphorylation of FAK and paxillin is mechanosensitive (integrin is a known mechanosensor) in smooth muscle [86, 88].

in smooth muscle [86, 88]. Cyclic strain promotes Pyk2 and FAK phosphorylation at focal adhesion sites in cells. The integrin-mediated activation of tyrosine kinases in turn modulates the functional status of downstream molecules such as paxillin and Hic-5 that are related to remodeling of the actin architecture [14].

Contractile stimulation also induces the recruitment of structural proteins that participate in the linkage of actin filaments to β-integrin in adhesion sites, which may strengthen the connection of actin filaments with the membrane. The cytoskeletal protein vinculin is recruited to the ILK-associated complex at adhesion sites [55]. Vinculin binds to talin via its head domain and to actin filaments via its tail domain. Contractile activation induces vinculin phosphorylation, which promotes the binding of vinculin to talin and actin filaments and tension development [29, 51].

α-Actinin is an actin cross-linking protein that also binds to integrin proteins. Stimulation with a contractile agonist induces rapidly the recruitment of α-actinin to the integrin-associated complexes of canine tracheal smooth muscle cells/tissues [52]. In the tracheal tissues, the recruitment of α-actinin is required for smooth muscle contraction but not for actin polymerization or myosin activation [52]. These observations demonstrate that disruption of the linkage of actin filaments to integrin-associated adhesions is sufficient to inhibit smooth muscle contraction [11, 14, 35] (Fig. 2).

#### Small GTPases and PAK in smooth muscle

Rho, Cdc42, and Rac are the members of the small GTPase Rho family. There is ample evidence that Cdc42 regulates actin polymerization in various cell types including smooth muscle cells [27, 28, 30, 89–92]. Activated Cdc42 binds to the GTP binding domain of N-WASP, inducing a conformational change and activating N-WASP, and triggering the nucleation of actin polymerization and actin filament branching [11, 27, 28, 73, 74]. In canine tracheal smooth muscle tissues, introduction of a dominant Cdc42 mutant depresses the activity of N-WASP concurrently with the decrease in actin polymerization and force development [11, 27, 28], suggesting that Cdc42-mediated actin assembly is essential for smooth muscle contraction.

Cdc42 and Rac are known to activate PAKs in mammalian cells [80, 93–96]. Although 6 isoforms have been found thus far, PAK1 is a major isoform in smooth muscle cells [80, 91–93, 95–98]. PAK has been shown to participate in the regulation of p38 MAP kinase in smooth muscle upon external activation [53, 93]. The activation of p38 MAP kinase may modulate the actin cytoskeleton and cell migration via heat shock protein 27 (HSP27) [14, 15, 53, 93]. *In vitro* biochemical studies have shown that unphosphorylated HSP25 (mouse and chicken homologs of HSP27) inhibits actin polymerization whereas phosphorylated HSP27 loses the ability to inhibit actin filament assembly [53, 94, 99, 100]. p38 MAP kinase may phosphorylate MAP kinase-activated protein (MAPKAP) kinase 2 (MK2), which subsequently catalyzes HSP27 phosphorylation and promotes actin polymerization and contraction [14, 53, 101] (Fig. 2).

PAK1 also regulates smooth muscle contraction by controlling the vimentin intermediate filament network [80, 91, 92, 94, 95, 97, 102]. Vimentin network undergoes phosphorylation at Ser-56 and spatial reorganization in smooth muscle cells/tissues in response to contractile stimulation, which is critical for smooth muscle force development [80, 95, 97, 102]. PAK1 deficiency reduces vimentin phosphorylation, reorientation of the vimentin network and smooth muscle contraction. Moreover, PAK1 is able to catalyze vimentin phosphorylation *in vitro* [80, 91, 92, 94, 95, 97, 102, 103]. Thus, PAK1 regulates smooth muscle force development in part by modulating vimentin phosphorylation and reorientation of the vimentin network.

Rho activation promotes actin stress fiber formation in cultured smooth muscle cells [104, 105], and actin polymerization in smooth muscle tissues during contractile stimulation [30]. In canine tracheal smooth muscle, contractile stimulation activates RhoA, which induces the independent recruitment of paxillin-vinculin complexes and FAK to cell adhesomes. Activated FAK induces the phosphorylation of paxillin, which remains bound to activated vinculin. Paxillin phosphorylation also facilitates the formation of a complex containing paxillin and Crk II with DOCK180 and PIX GEFs. This complex induces the activation of Cdc42, which in turn promotes the activation of N-WASP, which interacts with the Arp2/3 complex to induce actin polymerization in the cortical region of the smooth muscle cell [30] (Fig. 2).

#### Regulation of actin polymerization by histone deacetylase 8

Histone deacetylases (HDACs) are a family of enzymes that are originally identified as key regulators of histone deacetylation, nucleosome stability and gene transcription [106, 107]. The HDAC family members have long been thought to regulate nucleosomal histone acetylation solely. HDACs typically induce histone deacetylation and repress gene transcription [108]. However, recent studies suggest that some HDACs are present in the cytoplasm of nonmuscle cells, which has been implicated in regulating cell migration and microtubule dynamics [109, 110].

HDAC8 is localized both in the cytoplasm and the nucleus of mouse and human airway smooth muscle cells. HDAC8 is required for the contraction of smooth muscle tissues [111] and cultured smooth muscle cells [112]. In addition, cortactin is an actin-regulatory protein that undergoes deacetylation during migration of NIH 3 T3 cells [110]. Contractile stimulation induces cortactin deacetylation in mouse and human smooth muscle tissues. Knockdown or pharmacological inhibition of HDAC8 attenuates cortactin deacetylation, actin polymerization and contraction without affecting myosin activation. Furthermore, expression of a charge-neutralizing cortactin mutant inhibits contraction and actin dynamics during agonist activation [111]. Taken together, these results suggest a novel paradigm for the regulation of actin dynamics in smooth muscle: Upon contractile activation, HDAC8 induces cortactin deacetylation, which in turn promotes actin polymerization and the contraction in airway smooth muscle (Fig. 2).

#### Regulation by cofilin/ADF and vasodilator stimulated phosphoprotein (VASP)

Cofilin/ADF are members of a family of “actin-dynamizing proteins,” which are able to regulate the availability of actin monomers for actin filament assembly [11]. Cofilin binds to F-actin, severs actin filaments and provides more free barbed ends of actin filaments for nascent actin polymerization. Cofilin activity is regulated by its phosphorylation at Ser-3, which abolishes the ability of cofilin to bind to F-actin and thus inhibits its severing function [11, 45, 113].

In canine tracheal smooth muscle, contractile activation decreases cofilin phosphorylation at Ser-3, which is associated with force development [45]. Expression of an inactive phospho-cofilin mimetic (cofilin S3E) in the smooth muscle tissues inhibits endogenous ADF/cofilin dephosphorylation and actin polymerization. Expression of cofilin S3E in the tissues depresses tension development in response to ACh, but it does not influence myosin light chain phosphorylation. These observations verify the role of cofilin in regulating the availability of actin monomers for actin polymerization in smooth muscle [11, 45]. In addition, cofilin phosphorylation at Ser-3 is regulated by protein phosphatase 2B [11, 45].

Members of the Ena/VASP protein family can promote actin polymerization by elongating actin filaments. The elongation mechanism involves VASP oligomerization and its binding to profilin and vinculin. In tracheal smooth muscle, treatment with ACh or forskolin (an adenylyl cyclase activator) increases VASP Ser-157 phosphorylation [44]. VASP undergoes phosphorylation at Ser-157 in a biphasic manner in aortic smooth muscle during stimulation with phenylephrine [35]. Treatment with ACh but not forskolin triggers the formation of VASP-VASP complexes as well as VASP-vinculin and VASP-profilin complexes at membrane sites. VASP-VASP complex formation and the interaction of VASP with vinculin and profilin are inhibited by an inactive vinculin mutant, but VASP phosphorylation and membrane localization are unaffected [44]. Gunst et al. conclude that VASP phosphorylation at Ser-157 mediates its localization at the membrane, but Ser-157 phosphorylation and membrane localization are not sufficient to activate its actin polymerization activity (as in the forskolin case). The interaction of VASP with activated vinculin at membrane sites is essential for VASP-mediated actin polymerization (Fig. 2) [44].

### Adherens junctions and smooth muscle contraction

Adherens junctions are protein complexes that exist at cell–cell junctions in various cell types including epithelial cells, endothelial cells and muscle cells, which plays an essential role in intercellular connection and mechanotransduction [58, 114–116]. β-Catenin, a member of the armadillo family of proteins, is a key component of the cadherin-catenin complex in the plasma membrane [114]. β-Catenin is composed of an N-terminal head, an Arm (armadillo) domain and a C-terminal tail. The Arm domain of β-catenin binds to the cytoplasmic domain of cadherins; the extracellular domain of cadherins interacts with their counterparts of adjacent cells to form cell-cell contacts. The N-terminus of β-catenin interacts with actin filaments via the linker proteins such as α-catenin, vinculin and VASP [115, 117].

The cadherin-catenin complex is a mechanosensive and responsive structure [118]. The adherens junctions undergo reorganization in endothelial cells in response to tugging forces and thrombin treatment [118]. In keratinocytes, the engagement of the adherens junctions occurs upon chemical stimulation; the knockout of β-catenin disrupts the structural change [119]. The dynamic change of the adherens junctions may allow cells to adapt their mechanical property and intracellular signaling [118, 119].

**Fig. 3:**
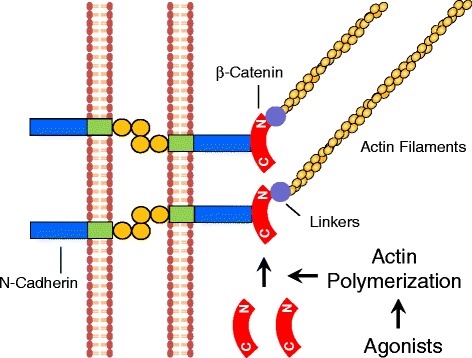
Novel mechanism for regulation of adherens junctions in smooth muscle. In addition to myosin activation, contractile agonists induce actin polymerization, which promotes the recruitment of β-catenin to N-cadherin. The increase in the protein-protein interaction may enhance the linkage of actin filaments to the adherens junctions, and promote the intercellular force transmission and smooth muscle contraction. C, C terminus; N, N terminus. Linkers, linker proteins such as α-catenin, vinculin and VASP

β-cateninis a necessary component of the cellular process that regulates smooth muscle contraction [58, 120]. However, β-catenin is not involved in the regulation of actin polymerization, myosin activation or contractile protein expression [58, 120]. Contractile stimulation promotes the recruitment of β-catenin to N-cadherin in human airway smooth muscle cells/tissues. This recruitment of β-catenin to N-cadherin is critical for smooth muscle contraction. Disruption of the protein-protein interaction by a β-catenin mutant attenuates smooth muscle force development [58]. More importantly, actin polymerization has a positive role in regulating the recruitment of β-catenin to N-cadherin. Together, these findings reveal a novel role of adherens junctions in smooth muscle contraction: Contractile stimulation promotes actin polymerization, which may increase the coupling of β-catenin with N-cadherin and facilitate intercellular mechanotransduction (Fig. 3).

## Role of actin-associated proteins in smooth muscle cell proliferation

Cell adhesion, spreading and migration are recognized as important cellular processes that control cell proliferation. Disruption of these processes ultimately impairs the proliferation in various cell types including smooth muscle cells [20, 21, 61, 121–125]. Actin-regulatory proteins are known to regulate these cellular functions [61, 93, 126]. Thus, actin-associated proteins are capable of regulating cell proliferation by controlling cell adhesion, spreading and movement. In this section of the review, I will focus on the roles of Abl and PAK1 in growth factor-mediated signaling and cytokinesis.

### Regulation of smooth muscle cell proliferation by Abl tyrosine kinase

Abl is activated in smooth muscle cells upon stimulation with platelet-derived growth factor (PDGF) and endothelin-1 [20, 21]. Endothilin-1 receptor is a member of G protein-coupled receptor (GPCR) family whereas PDGF receptor belongs to the family of tyrosine kinase-containing receptors. Both GPCR and tyrosine kinase receptors play an important role in the regulation of smooth muscle cell functions [22, 127]. In rat vascular smooth muscle cells, activation of GPCR by agonists induces phosphorylation of Src at Tyr-416, which in turn triggers the activation of Abl [21, 47, 67]. Similarly, cellular challenge with PDGF is able to activate Src, which subsequently mediates phosphorylation of Abl in mammalian cells [21, 128].

**Fig. 4:**
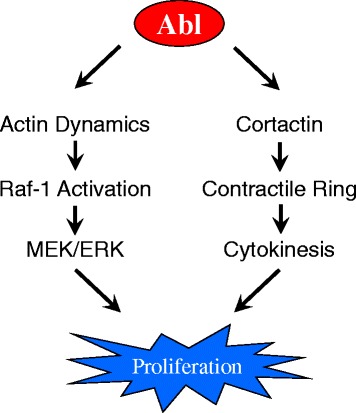
Regulation of MAPK pathway and cytokinesis by Abl. Upon the binding of ligands to growth factor receptors, Abl promotes Raf-1 activation by controlling actin dynamics, which in turn modulates the activation of MEK and ERK. In addition, Abl is recruited to the midzone during cytokinesis, which mediates cortactin phosphorylation. Phosphorylated cortactin promotes F-actin assembly, which facilitates contractile ring formation and cytokinesis

Abl is necessary for the proliferation in smooth muscle cells in response to activation with growth factors [20, 21]. Upon the binding of ligands to growth factor receptors, Raf-1 kinase is translocated to the plasma membrane, which subsequently activates Raf-1. Activated Raf-1 phosphorylates MEK1/2 (MAPK kinase), which in turn phosphorylates and activates ERK1/2 and promotes cell proliferation [22, 23]. In human airway smooth muscle cells, PDGF activation induces an increase in the association of Raf-1 with cytoskeletal actin. Inhibition of actin polymerization by Abl knockdown or latrunculin A attenuates the interaction of Raf-1 with F-actin and Raf-1 redistribution during PDGF stimulation. Furthermore, Abl knockdown inhibits the PDGF-induced MEK and ERK1/2 phosphorylation and the proliferation in human airway smooth muscle cells (Fig. 4) [20, 21]. However, Abl does not affect AKT activation in smooth muscle cells upon growth factor stimulation [20, 21]. Abl regulation of ERK1/2 phosphorylation has also been reported in nonmuscle cells (e.g., fibroblasts); Abl appears to regulate SHP-2 tyrosine phosphatase, which subsequently modulates ERK1/2 activation in fibroblasts [129].

Abl also regulates smooth muscle cell proliferation by controlling cytokinesis, a critical step of cell division [125]. Abl is localized in the contractile ring of human airway smooth muscle cells. Knockdown or inhibition of Abl attenuates cytokinesis in smooth muscle cells [125]. Cortactin is a tyrosine-phosphorylated protein that has been implicated in the regulation of actin filament assembly [75, 76]. Phosphorylated cortactin is also found in the contractile ring. Abl knockdown or inhibition attenuates cortactin phosphorylation in the midzone and contractile ring formation. Furthermore, the expression of a nonphosphorylatable cortactin mutant diminishes cytokinesis. Thus, these results uncover a novel mechanism: Abl is recruited to the equator during cytokinesis, which mediates cortactin phosphorylation. Phosphorylated cortactin promotes actin filament assembly, which facilitates contractile ring formation and cytokinesis (Fig. 4) [125].

### Role of PAK1 in growth factor-mediated signaling

PAK1 is phosphorylated and activated in rat aortic smooth muscle cells upon stimulation with growth factors and angiotensin II [130, 131]. Furthermore, PAK1 activity is necessary for rat aortic smooth muscle cell proliferation [24]. In addition, PAK1 is required for mitotic progress in nonmuscle cells [132].

PAK1 is likely to regulate smooth muscle cell proliferation by modulating ERK1/2 activation [133–135]. In response to cell adhesion to fibronectin, PAK mediates MEK1/2 phosphorylation at Ser-298, which subsequently promotes phosphorylation of Ser-218/Ser-222 and activates MEK1/2 [135]. Similarly, PAK1 is able to regulate ERK1/2 activation during migration of macrophage [133]. However, these studies have been performed in vascular smooth muscle cells or nonmuscle cells. Future studies are required to evaluate whether PAK1 has similar or distinctive role in airway smooth muscle cell proliferation.

## Role of cytoskeleton-regulatory proteins in AHR and airway remodeling

### Critical role of Abl in AHR and airway remodeling *in vivo*

Asthma is characterized by AHR and airway remodeling, which are largely due to increased airway smooth muscle contractility [1–5] and cell proliferation [7, 8]. As described earlier, the function of Abl in smooth muscle contraction and cell proliferation *in vitro* has been documented. Until recently, the role of Abl tyrosine kinase in the pathogenesis of AHR and airway remodeling has been elusive. In a recent study [5], the expression of Abl is upregulated in airway smooth muscle tissues of an animal model of asthma and in asthmatic human airway smooth muscle cells. Conditional knockout of Abl in smooth muscle attenuates the methacholine-induced airway reactivity in animals sensitized and challenged with ovalbumin. Intranasal instillation with the Abl inhibitors imatinib and GNF-5 also inhibits airway resistance in the diseased animals. These results are supported by a previous study by others [64]. Furthermore, conditional knockout of Abl or treatment with the Abl inhibitors suppresses the agonist-induced airway smooth muscle hyperreactivity *in vitro*. The allergen-induced smooth muscle mass and cell proliferation are also reduced in the airways of Abl knockout mice or the inhibitor-treated mice exposed to the allergen [5]. Taken in sum, these findings demonstrate a pivotal role of Abl in the allgern-induced AHR and airway remodeling *in vivo*.

In response to allergic sensitization and challenge, inflammatory cells enter into the lungs and cytokine/chemokine levels are increased in the bronchoalveolar space of asthmatic patients and animal models [4, 136, 137]. Because airway smooth muscle cells have ability to secrete cytokines *in vitro* [138], the effects of Abl knockout in smooth muscle on airway inflammation was also evaluated. Conditional knockout of Abl does not affect the increase in inflammatory cell numbers, IL-13 and CCL2 in animals sensitized and challenged by the allergen. On the contrary, treatment with imatinib and GNF-5 reduces the allergen-induced increase in inflammatory cell numbers, and levels of IL-13 and CCL2 [5]. The results suggest that global inhibition of Abl diminishes airway inflammation in chronic asthma, which is consistent with the findings that Abl may regulate migration and synthetic functions of immune cells *in vitro* [139–142].

There is a need to identify new biological target for the development of new therapy to treat asthma. As mentioned above, preclinical studies suggest that Abl has a critical role in the pathogenesis of AHR, airway remodeling and inflammation [5, 64]. In addition, clinical studies suggest that the Abl inhibitor imatinib is effective to lessen the clinical symptoms of patients with pulmonary arterial hypertension [65, 66], a disease that is also involved in abnormal smooth muscle cell contraction and proliferation. Thus, these findings support the concept that Abl may be a novel target for the development of new therapy to treat asthma.

### Role of PAK1 in AHR in vivo

The potential role of PAK1 in the development of AHR was described in a recent study [6]. Tepper et al. demonstrate that inhibiting the activity of p21-activated protein kinase is an effective approach for reducing AHR *in vivo*. PAK1 knockout mice display significantly lower airway resistance *in vivo*, and the aerosol administration of a synthetic small molecule PAK inhibitor also reduces AHR in mice. Furthermore, isolated tracheal segments from PAK1^−/−^ mice exhibit reduced contractile response *in vitro*, indicating that the decrease in airway reactivity observed *in vivo* in PAK1^−/−^ mice results from decreased airway smooth muscle contraction [6].

However, they did not detect differences in the structure of the airways and lung parenchyma in PAK1^−/−^ and wild type animals [6]. Decreased airway responsiveness does not appear to result from other factors that can alter airway responsiveness. These observations provide evidence that the reduction of PAK activity inhibits airway resistance *in vivo* and that this effect is caused by a reduction in the contractility of the airway smooth muscle. Furthermore, PAK inhibition is also able to attenuate the contraction in human bronchial tissues, which suggests that this approach could be effective in humans *in vivo* also.

## Conclusions and perspectives

Exploring the functions and regulation of actin-associated proteins in smooth muscle is a new trend in smooth muscle physiology and asthma research. There is a wealth of evidence that actin polymerization is a key cellular process that controls smooth muscle contraction. Abl tyrosine kinase regulates actin cytoskeletal remodeling by controlling several downstream effectors including Abi1, cortactin and GMF-γ. Abl also has a role in regulating smooth muscle cell proliferation. Investigating potential roles of Abl in other smooth muscle functions is an interesting topic. There is emerging evidence that actin polymerization is modulated by protein deacetylation and that actin polymerization affects adherens junctions and cell-to-cell mechanotransduction. Understanding how protein acetylation/deacetylation and adherens junction assembly are regulated will be important directions in this research area. Abl and PAK1 are involved in the pathogenesis of asthma, suggesting that they are potential biotargets for the development of new treatment of asthma. Elegant technologies have been employed to unveil the roles of the integrin-associated complex and other actin-regulatory proteins in smooth muscle. However, there is not much information on whether these complex interactions are involved in asthma pathology. Filling this knowledge gap will enhance our understanding of asthma pathology.

## Abbreviations

Abi1: Abl interactor 1
Abl: Abelson tyrosine kinase
ACh: Acetylcholine
ADF: Actin-depolymerization factor
AHR: Airway hyperresponsiveness
CAS: Crk-associated substrates
ERK1/2: Extracellular signal-regulated kinase1/2
FAK: Focal adhesion kinase
GMF-γ: Glia maturation factor-γ
GPCR: G protein-coupled receptor
HDACs: Histone deacetylases
HSP: Heat shock protein
IL: Interleukin
ILK: Integrin-linked kinase
MAPK: Mitogen-activated protein kinase
MK2: MAP kinase-activated protein (MAPKAP) kinase 2
N-WASP: Neuronal Wiskott-Aldrich Syndrome Protein
PAK: p21-activated kinase
PDGF: Platelet-derived growth factor
Pfn-1: profilin-1
VASP: Vasodilator stimulated phosphoprotein
